# Quetiapine and Wolff-Parkinson-White Syndrome

**DOI:** 10.1155/2020/6633385

**Published:** 2020-12-11

**Authors:** Michael Chen, Hassaan Gomaa, Alonso Cortez-Resendiz, Christopher Martin, Andrew Francis, Alfredo Bellon

**Affiliations:** ^1^Department of Psychiatry, Lehigh Valley Health Network, 2545 Schoenersville road, Bethlehem PA 18017, USA; ^2^Department of Psychiatry and Behavioral Health, Penn State Hershey Medical Center, 500 University Drive P.O. Box 850. Mail Code R130. Hershey, PA 17033-0850, USA; ^3^Department of Psychiatry, Thomas Jefferson University, Philadelphia, PA, USA; ^4^Department of Pharmacology, Penn State College of Medicine, Penn State Hershey Medical Center, Hershey, PA, USA

## Abstract

Quetiapine is occasionally associated with cardiovascular adverse effects such as QTc prolongation. QTc prolongation is a side effect that requires monitoring in order to avoid more serious cardiac complications. One particular understudied area is the potential for antipsychotics to elicit electroconduction abnormalities in patients with Wolff-Parkinson-White (WPW) Syndrome. In the present report, we describe a case of quetiapine overdose in a patient with WPW.

## 1. Introduction

Wolff-Parkinson-White (WPW) Syndrome is a cardiac condition deviation characterized by an accessory pathway that bypasses the atrioventricular (AV) node resulting in abnormally fast ventricular contractions. Specific electrocardiogram (EKG) findings of WPW include delta wave, widened QRS complex (>110 ms), shortened PR interval (<120 ms), and inverted T waves [1]. Although generally asymptomatic [2], the most common clinical symptom is “palpitations of the heart [3].” In rare, severe cases, WPW has been linked to Torsades de pointes and sudden cardiac death [4]. While the pathophysiology of WPW is well established and studied, the effects of antipsychotics on patients with WPW remain unknown.

Other cardiac consequences of antipsychotic medications have been well characterized, including QTc prolongation [5]. Specifically, quetiapine is well known to have QTc prolonging effects and has been linked to dangerous conditions such as Torsades de pointes [6]. However, the potential effects of quetiapine on patients with WPW have not been described. Therefore, here, we report the consequences of a quetiapine overdose in a patient with WPW.

## 2. Case Report

**Figure 1:**
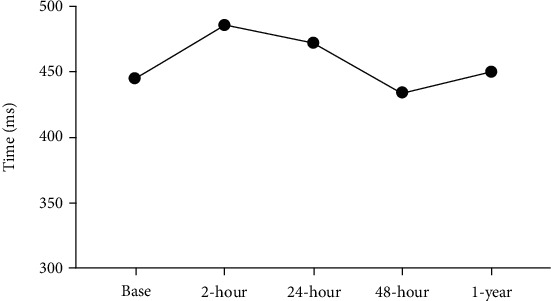
Effects of quetiapine on QTc on a patient with Wolff-Parkinson-White (WPW) Syndrome. This graph represents changes in the QTc interval expressed in milliseconds at various points in time including at baseline, 2, 24, and 48 hours postoverdose and then finally a year after.

A 30-year-old female with a past medical history significant for WPW and a psychiatric history of major depressive disorder, posttraumatic stress disorder, borderline personality disorder, and several suicide attempts presented to the Emergency Department (ED) with complaints of decreased energy, swollen lymph nodes, weight loss (approximately 13.5 kg), and mild abdominal pain. Subsequent interviews revealed that while waiting for the results of her evaluation, the patient overdosed on 1200 mg of trazodone, 1200 mg of quetiapine, and 900 mg of venlafaxine, as a suicide attempt. Two hours postoverdose, the patient began to exhibit a prolonged QTc interval of 486 ms which contrasted with her QTc baseline of 445 ms (Figure 1). Of note, no delta wave was appreciated in the EKG. Her PR interval was 160 ms at baseline and 146 ms 2 hrs after (Table 1). Baseline blood pressure was noted at 114/70, but 2 hours postoverdose, the patient developed hypotension of 86/45 mmHg. In addition, the patient complained of palpitations with a heart rate ranging from 83 to 97. All psychiatric medications were discontinued, and intravenous fluids were provided. Forty-eight hours after discontinuation of her medication, the patient's EKG normalized including her QTc interval (QT/QTc of 414/434) (Figure 1), and her PR interval was at 160 ms (Table 1). At this point, the patient was medically cleared and transferred to our inpatient unit.

On the inpatient unit, the patient was slowly reintroduced to her psychiatric medications. On day 1, the patient was started on quetiapine 100 mg at bedtime, trazodone 100 mg at bedtime, and venlafaxine 150 mg in the morning. This was gradually raised to a maximum dose of quetiapine100 mg in am and 300 mg at bedtime. Venlafaxine was increased to 225 mg daily and trazodone remained at 100 mg at bedtime. Apart from mild sedation, the patient reported no symptoms or side effects from medications such as sensation of rapid, fluttering, or pounding heartbeats (palpitations); dizziness or lightheadedness; or shortness of breath. The patient was discharged after 11 days of inpatient psychiatric treatment.

After discharge, maintenance treatment consisted of 300 mg of quetiapine, 100 mg of trazodone, and 225 mg of venlafaxine. A year later, the patient was seen in the ED for chest palpitations, pain, and dizziness. Her initial EKG was significant for heart rate of 109 beats per minute, QTc of 450 ms, and PR interval of 160 ms. Twenty-four hours later her EKG showed a QTc interval of 457 ms, PR interval of 170 ms, and heart rate of 89 bpm (Figure 1). Of note, delta waves were not appreciated on either EKG. Initially, the patient was worked up for chest pain via 3 sets of troponins, EKG, and stress test. All test results came back negative. Her symptoms resolved within 24 hours with no changes in medication, and the patient was discharged without further issues.

## 3. Discussion

In recent years, special consideration has been placed on the QTc prolonging side effects of various psychotropic medications, particularly, typical antipsychotics such as haloperidol [7], droperidol [8], and atypical antipsychotics such as olanzapine, ziprasidone, and risperidone [9]. But very little is known about the effects antipsychotics may have on patients with WPW. We have recently reviewed all published information currently available [10], which mostly comes from case reports, some providing conflicting results. For example, olanzapine has been shown to have varying levels of safety in WPW patients. In one case a dosage of 5 mg/day was shown to cause QTc prolongation from 390 to 466 ms [11]. In another case, a dosage of 7.5 mg/day was shown to have no effect on QTc intervals [12]. On the other hand, data on risperidone is more consistent. In two separate cases, risperidone at 1 mg BID induced palpitations, QTc prolongation, shortened PR intervals, and induction of a delta wave on EKG [9, 13]. To our knowledge, there is no published data on the effects of quetiapine on WPW.

Quetiapine is a dibenzothiazepine derivative that is FDA-approved to treat schizophrenia and bipolar disorder. Compared to typical antipsychotics, quetiapine is unique in that it has transient antagonist effects on dopamine 2 receptors and, as a result, lower risk for extrapyramidal symptoms [14]. At the same time, when compared to typical antipsychotics, quetiapine has an affinity for muscarinic receptors resulting in anticholinergic side effects such as orthostatic hypotension and transient sedation [15].

The general medical effects of quetiapine overdose have been documented in several previous case studies. Most overdose cases result in exaggeration of the anticholinergic side effects including tachycardia, hypotension, and sedation. However, several other symptoms have been documented in severe overdoses. One report in a 26-year-old woman who ingested over 10,000 mg of quetiapine, resulted in rapid deterioration of mental status and required intubation [16]. Another case was a 19-year-old man who ingested 9,600 mg of quetiapine, leading to QTc prolongation [17]. A third publication described a 31-year-old female who ingested 2,000 mg of quetiapine and then became comatose requiring intubation. QTc prolongation was also observed [5].

In our case, our patient had an overdose of 1,200 mg that resulted in typical anticholinergic side effects of hypotension and sedation. Initially, the patient presented with concerning symptoms of QTc prolongation. However, despite having an extra electrical pathway, the patient did not develop any further cardiac complications such as arrhythmia. In fact, discontinuation of quetiapine led to recovery within 48 hours. Furthermore, after reintroduction of quetiapine to therapeutic levels, the patient did not present any clinical symptoms associated with WPW such as palpitations or shortness of breath. The only symptoms that the patient expressed were mild fatigue, which can be attributed to the anticholinergic effects of quetiapine and mild QTc prolongation. Of note, the side effect of fatigue resolved after slightly lowering her quetiapine dose. Her QT interval also returned to baseline.

Most importantly, the patient was discharged on a therapeutic dose of quetiapine with no cardiac complications. This was confirmed upon reevaluation 1 year after discharge in which the patient initially reported some symptoms that could be related to WPW. While the patient did report palpitations, the EKG demonstrated a QTc interval similar to baseline. A repeat EKG 24 hours later remained at baseline. Thus, it is evident that 1 year of therapeutic dosage of quetiapine did not affect the patient's WPW.

It also has to be considered that the patient was concomitantly taking trazodone and venlafaxine. While some studies have shown a slight increase in QTc interval with trazodone [18] and venlafaxine [19], this side effect is less frequently observed than when quetiapine is administered. The combination of quetiapine with trazodone, especially considering that the patient overdosed on 2000 mg of trazodone, could have elicited significant QTc prolongation and even Torsades. Serious cardiac arrhythmias have been documented in cases of trazodone and venlafaxine overdose or when combined with other QTc prolonging medications [20].

In conclusion, to our knowledge, this is the first case reporting the potential effects of quetiapine, trazodone, and venlafaxine in an individual with WPW, and no serious cardiac complications were encountered even after a year of follow-up. But these medications cannot be considered entirely safe for patients with WPW until further research is developed on this topic.

## Figures and Tables

**Table 1:** EKG parameters at baseline and several time points after quetiapine overdose.

	QT	QTc	PR interval	Pulse
Baseline	358 ms	445 ms	160 ms	93 BPM
2-hour postintoxication	416 ms	486 ms	146 ms	82 BPM
24-hour postintoxication	404 ms	472 ms	180 ms	82 BPM
48-hour postintoxication	414 ms	434 ms	160 ms	66 BPM
1-year postintoxication	344 ms	450 ms	170 ms	89 BPM

## Data Availability

All data used for this work is included in the manuscript.
